# Treatment Enhances Betweenness Centrality of Fronto-Parietal Network in Parkinson’s Patients

**DOI:** 10.3389/fncom.2022.891384

**Published:** 2022-05-26

**Authors:** Qing Liu, ZhongYan Shi, Kexin Wang, Tiantian Liu, Shintaro Funahashi, Jinglong Wu, Jian Zhang

**Affiliations:** ^1^Laboratory for Brain Science and Neurotechnology, School of Life Sciences, Beijing Institute of Technology, Beijing, China; ^2^Advanced Research Institute of Multidisciplinary Science, Beijing Institute of Technology, Beijing, China; ^3^Research Center for Medical Artificial Intelligence, Shenzhen Institutes of Advanced Technology, Chinese Academy of Sciences, Shenzhen, China; ^4^School of Mechatronical Engineering, Beijing Institute of Technology, Beijing, China

**Keywords:** Parkinson’s disease, resting state-fMRI, functional connectivity, fronto_parietal network, graph theory

## Abstract

Previous studies have demonstrated a close relationship between early Parkinson’s disease and functional network abnormalities. However, the pattern of brain changes in the early stages of Parkinson’s disease has not been confirmed, which has important implications for the study of clinical indicators of Parkinson’s disease. Therefore, we investigated the functional connectivity before and after treatment in patients with early Parkinson’s disease, and further investigated the relationship between some topological properties and clinicopathological indicators. We included resting state-fMRI (rs-fMRI) data from 27 patients with early Parkinson’s disease aged 50–75 years from the Parkinson’s Disease Progression Markers Initiative (PPMI). The results showed that the functional connectivity of 6 networks, cerebellum network (CBN), cingulo_opercular network (CON), default network (DMN), fronto-parietal network (FPN), occipital network (OCC), and sensorimotor network (SMN), was significantly changed. Compared to before treatment, the main functional connections were concentrated in the CBN after treatment. In addition, the coefficients of these nodes have also changed. For betweenness centrality (BC), the FPN showed a significant improvement in treatment (*p* < 0.001). In conclusion, the alteration of functional networks in early Parkinson’s patients is critical for clarifying the mechanisms of early diagnosis of the disease.

## Introduction

Parkinson’s disease (PD) is a multi-system neurodegenerative disease that commonly affects middle-aged and elderly people. The main pathological feature of PD is the degeneration and death of substantia nigra dopamine neurons (Bene et al., 2009; Wu and Hallett, 2013). The clinical features of PD are movement disorders such as rigidity, resting tremor, bradykinesia, and gait disturbance (Bene et al., 2009; Colosimo et al., 2010). Currently, due to the clinical features of PD, resting-state functional magnetic resonance imaging (rs-fMRI) techniques are used in clinical diagnosis to study the pathogenesis of PD.

Rs-fMRI is an emerging non-invasive method combining functional, imaging, and anatomical information to detect spontaneous neural activity at baseline. Magnetic resonance imaging, as a non-invasive examination method, will be used for long-term medical examinations, while functional magnetic resonance imaging, as a magnetic resonance imaging method that reflects the level of blood oxygen in the brain, can effectively and real-time reflect the state of brain activity (Wong et al., 2021). In addition, rs-fMRI can non-invasively detect various functional areas of the brain, so it can be used for long-term brain research (Emiliano et al., 2012). FMRI includes task-state functional magnetic resonance imaging and rs-fMRI. Rs-fMRI refers to subjects remaining still, eyes closed, not thinking, not instructing, or assigning tasks during the examination (Crossley et al., 2014). Resting-state imaging is a state in which the subject remains awake, not receiving any external stimuli, and performing any advanced tasks. Task state imaging is that subjects complete some specific tasks during the inspection process, such as observing pictures, finger movements, airflow tactile stimulation, etc. Rs-fMRI mainly detects spontaneous fluctuations of unstimulated blood oxygen level-dependent effects. It shows the spontaneous activity of neurons in the resting state (Gold et al., 2007; Goetz et al., 2010). Compared to task-state imaging, rs-fMRI has many advantages: no complex tasks, easy patient coordination, and high experimental reproducibility (Don et al., 2021; Niu et al., 2021). Furthermore, it reduces the impact of specific tasks on subjects (Shalchy and Asemani, 2015; Sair et al., 2016). Rs-fMRI can study different neural networks simultaneously. It improves the success rate of diagnosing disease-related connections. Rs-fMRI technology has been very mature in the application of mental diseases, such as PD, Alzheimer’s disease and depression (Yan et al., 2018, 2019; Yang et al., 2018, 2020; Liu et al., 2022). Now, resting-state-based brain functional network studies have identified some intrinsic networks in the brain, such as sensorimotor network (SMN) (Lagana et al., 2020; Caspers et al., 2021; Palmer et al., 2021), fronto-parietal network (FPN) (Engels et al., 2018; Boon et al., 2020), default network (DMN) (Rektorova, 2014; Putcha et al., 2015), etc. For PD, the main research point is to focus on changes in resting brain networks in patients with Parkinsonian movement disorders (Liu et al., 2021).

As an important organ of the human body, the complex structure and function of the brain are the focus of scholars’ research. The topology between neurons, or clusters of neurons, forms a complex brain network that determines how the entire brain works. Graph theory is an important tool for describing network characteristics and is widely used to study the topological properties of structural and functional networks in the human brain (Medani et al., 2010; Dehmer et al., 2017). It provides a complex brain network model and helps to better understand the connections between network structures. Graph theory can be used to examine the connections between brain functional connectivity and information integration and human behavior, as well as to examine the impact of neurological diseases on brain network structure (Medani et al., 2010). In recent years, based on the complex network theory of graph theory, researchers have found that functional brain networks constructed by rs-fMRI have many important topological properties. For example, degree centrality (DC) shows many connections between cognition and behavior. The complex functional network of the brain is affected by brain diseases such as schizophrenia, Alzheimer’s disease and PD (Xie et al., 2019; Li et al., 2020; Pei et al., 2020). Graph theory is a great way to uncover the impact of these diseases on brain function. In addition, there are marked functional abnormalities in the brain networks of PD. These abnormalities are concentrated in the SMN and FPN (Dung and Thao, 2018; Chung et al., 2019). The functional connectivity and topological properties of these networks change as the disease progresses. Combining rs-fMRI analysis with graph theory analysis is beneficial to study the important role of specific brain networks in PD.

At present, the mechanism of changes in cerebral cortex function is a hot issue in the research of central nervous system diseases, but there are few reports on the mechanism of changes in cerebral cortex function related to PD. In previous studies, some scholars have used functional imaging equipment such as PET and MRI to prove that there are metabolic and functional disorders in the frontal and parietal cortex of PD patients (Monchi et al., 2004; Beyer and Aarsland, 2008; Li et al., 2018). However, current research on PD has focused on the nigrostriatal system (Dung and Thao, 2018). Most of the migratory functional areas of the cerebral cortex are located in the frontal and parietal cortex, and most of the connective fibers between the basal ganglia and the cortex project to this area simultaneously. Moreover, the cortex of the frontal and parietal lobes plays an important role in the development of movement as direct and indirect feedback channels for information processing. A large number of studies have shown that under rs-fMRI, patients with PD have reduced functional connectivity in the sensorimotor area, frontal and parietal networks (Lee et al., 2012; Baek et al., 2014), and Parkinson’s drug treatment can improve and enhance the functional connectivity of the FPN. Changes in the functional connectivity of the FPN can be regarded as an important feature of PD. Therefore, studying the functional changes in this region will help people to understand the pathogenesis of this disease more deeply.

This study aims to explore the pathogenesis of PD, find the key networks and brain regions for early PD treatment, and provide a scientific basis for clinical treatment of PD. We analyzed rs-fMRI data from the Parkinson’s progression markers initiative of 27 patients aged 50 to 75 years with early-stage PD. In this study, by reconstructing the cerebellum network (CBN), cingulo_opercular network (CON), DMN, FPN, occipital lobe network (OCC), SMN and other brain functional networks, to explore the par Brain changes in Kinson’s disease. At the network level, compared to before treatment, we analyzed improvements in functional connectivity and topological properties of brain functional networks in all PD patients. Specific steps are as follows. First, compared to before treatment, functional connectivity and topological properties of all brain regions were analyzed by paired *t*-test to find improved brain regions. Second, the functional connectivity and topological properties of individual networks were obtained by weighted averaging of the functional connectivity and topological properties of the brain regions included in the network. The same paired *t*-test was applied. Finally, brain regions and networks with significant differences before and after treatment were selected and Pearson-correlated with dyskinesia scores.

## Materials and Methods

### Participants

All rs-fMRI data and T1 data were downloaded from PPMI.^1^ The PPMI dataset is a comprehensive observational international multicenter study to identify biomarkers of PD progression, improve understanding of disease etiology and progression, and provide biomarkers to improve PD treatment efficacy. Data was downloaded from PPMI database on September 21, 2020.

In this dataset, patients were screened by inclusion criteria: (1) aged between 50 and 75 years; (2) with asymmetric resting tremor or asymmetric bradykinesia, or both of these symptoms, including bradykinesia, resting Tremor, and rigidity; (3) PD diagnosed within 2 years of study enrollment; (4) Hoehn and Yahr stage (HY) I or II at enrollment. Prior to the start of the study, each study site participating in the PPMI study was approved by the Human Experimentation Ethics Standards Committee, and written informed consent was obtained from all participants. Disease staging was assessed using the Hoehn and Yahr staging score, disease severity was assessed using the Unified PD Rating Scale III (UPDRSIII), and overall cognitive function was assessed using the Mini Mental State Examination (MMSE). Details of the study methodology have been published elsewhere and are available on the PPMI website. After exclusion of poor quality neuroimaging data, 27 PD patients (61.16 ± 7.80 years) were finally included in the analysis. Prior to the study, each PPMI subject was approved by the Human Trials Ethics Committee and signed an informed consent form.

### Neuroimaging Data Preprocessing

Based on the MATLAB software platform, the DPABI toolbox was used to preprocess the rs-fMRI data. The steps are as follows. First, the first 10 time points were removed to improve the signal-to-noise ratio. Second, slice time and head motion was corrected for differences between scan layers. Third, functional data were normalized to Montreal Neurological Institute (MNI) space based on anatomical images (T1-weighted images) of each subject to obtain time-series signals of various regions of the brain. Fourth, spatially smooth the data. Fifth, global mean signal intensities were normalized across operations, filtered through a temporal bandpass (0.01–0.08 Hz), and detrimental covariates (head motion, ventricle, white matter and CSF signals, and whole-brain signals) were regressed to improve movement Correction of related artifacts. Finally, the brain was divided into 160 regions using the Dosenbach_Science_160 ROIs atlas (Dos160) (Dosenbach et al., 2010). The average time signal series of each brain region was extracted, and the Pearson correlation coefficient of each pair of brain region time series was calculated as the functional connectivity strength of the brain region. The correlation coefficients were converted to a normal distribution using Fisher’s Z transformation (Ewing-Cobbs et al., 2006). The calculation results are represented by a 160 × 160 correlation coefficient matrix (Nandhagopal et al., 2009; Gollo et al., 2015).

### Functional Network Construction

A network consists of edges and nodes. The entire cerebral cortex is divided into 160 regions according to the Dosenbach_Science_160 ROIs atlas. For individual networks, 160 distinct brain regions in the Dos160 graph are defined as nodes, and reconstructed fiber tracts connecting brain regions represent edges. The connectivity features of individual subjects are represented by the strength of connectivity between regions of interest. The average signal of the ROI was calculated by averaging the preprocessed bold signal over all vertices within the ROI. Then, the connectivity between the two ROIs is estimated using Pearson correlation and converted to *Z*-values using Fisher z-transform (Sung et al., 2016).

To elucidate changes in each functional network, these cortical connections were grouped according to six functional networks, including the CBN, the CON, the DMN, the FPN, the OCC, and the SMN. First, we performed independent paired *t*-test on the functional connectivity of all regions of interest before and after treatment to select brain regions with significant differences (Kubler et al., 2019). Based on selected brain regions, we calculated average functional connectivity values for each network. Second, we used GRETNA^2^ to calculate global network parameters, including network efficiency, local efficiency, and small-world properties (Stam et al., 2007), and node network parameters, including each participant’s BC, DC, Node Clustering Coefficient (NCp), Node Efficiency (Ne), Node Local Efficiency (NLe), Node Shortest Path (NLp) (Zhang et al., 2012; Yamamoto et al., 2017). Third, brain regions with significant differences were selected for subsequent analysis. Finally, the same analysis steps were applied at the network level.

### Statistical Analysis

All statistical analyses were performed using the Social Sciences Statistical Package 23.0.^3^ To more intuitively compare the changes in each brain region before and after treatment, we used paired *t*-test to compare the functional connectivity and nodal parameters of patients before and after treatment. We applied multiple comparison false detection rate (FDR) correction, *Q* < 0.05 (Benjamini and Hochberg, 1995), to obtain brain regions with significant changes (Engels et al., 2018). The functional connectivity and topological properties of selected brain regions were Pearson-correlated with the motor scale UPDRSIII to determine whether this brain region was strongly associated with motor symptoms. On the network side, the number of brain regions in each brain network was recorded. The average value of the topological properties in the brain network is calculated instead of the total value as the topological properties of this brain network to avoid the influence of network size.

## Results

### Significant Differences in Functional Connectivity After Treatment

**FIGURE 1:**
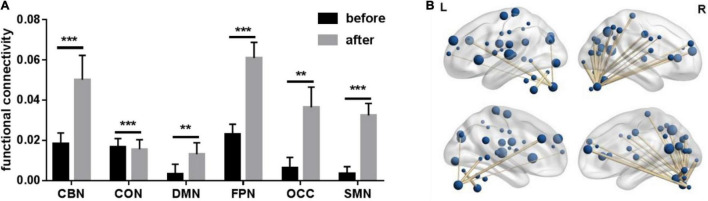
Paired *t*-test results of average functional connectivity of six networks before vs. after treatment. CBN, cerebellum network; CON, cingulo_opercular network; DMN, default network; FPN, fronto-parietal network; OCC, occipital lobe network; SMN, sensorimotor network. ** Represents a significance level of *p* < 0.01, *** represents a significance level of *p* < 0.001. **(A)** Paired *t*-test bar chart of average functional connections of six networks before vs. after treatment. **(B)** Functional connections with significant differences after treatment.

Table 1 lists the demographic characteristics of the 27 PD patients. We calculated functional connectivity before and after treatment in 27 PD patients. The functional connectivity with significant differences in each network was selected and then averaged as the functional connectivity of each network. Paired *t*-test was performed on functional connectivity of each network before and after treatment. All results are FDR corrected. As shown in Figure 1, including CBN (*p* < 0.001), CON (*p* < 0.001), DMN (*p* = 0.004), FPN (*p* < 0.001), OCC (*p* = 0.001), and SMN (*p* < 0.001), all six networks were significantly different. Furthermore, the functional connectivity of CBN, DMN, FPN, OCC, and SMN was significantly enhanced after treatment compared with before treatment, while the opposite was true for CON.

**TABLE 1:** Basic demographic characteristics and global network parameters.

Feature	PD (pre)	PD (post)	Group comparisons (statistical significance)
**Demographics**			
Age (years)	61.16 ± 7.80	63.16 ± 5.78	*p* < 0.001***
Gender	14M/13F	14M/13F	N/A
Weight (kg)	80.81 ± 17.71	79.53 ± 16.82	*p* = 0.342
HY	1.44 ± 0.51	1.82 ± 0.48	N/A
**Motor**			
MDS-UPDRSII	5.08 ± 2.32	7.00 ± 2.53	*p* = 0.264
MDS-UPDRSIII	17.07 ± 6.22	20.33 ± 10.34	*p* = 0.003**
**Non-motor**			
MDS-UPDRSI	9.12 ± 6.23	9.04 ± 4.53	*p* = 0.168

MDS-UPDRS, Movement Disorders Society-Unified Parkinson’s Disease Rating Scale. **Represents a significance level of p < 0.01, *** represents a significance level of p < 0.001.

The functional connectivity with significant difference (*p* < 0.05) before and after treatment was selected, and then the difference in functional connectivity was Pearson correlated with the difference in motor symptom scale (UPDRSIII) before and after treatment. Finally, 82 edges with significant correlation were selected (see Figure 1). Notably, Figure 1 visually shows that relevant functional connectivity is clustered in the cerebellum.

### Analysis of Node Network Parameter

We performed independent paired *t*-test on 6 different node network parameters for each ROI (160 regions of interest) in PD patients before and after treatment, including BC, DC, NCp, Ne, NLe, NLp.

**FIGURE 2:**
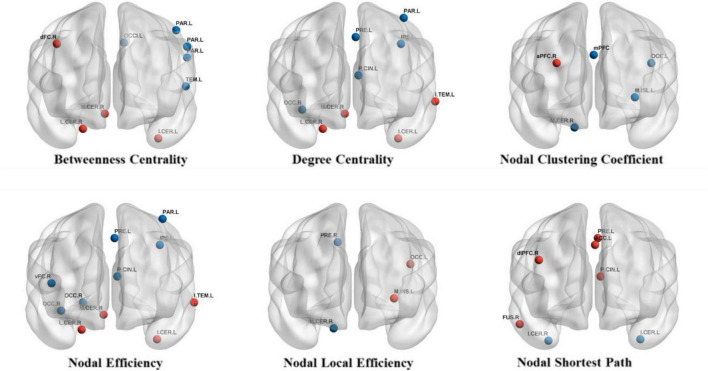
Brain regions with significant changes in nodal parameters. The red circles represent brain regions with significantly increase nodal parameter after treatment and the blue circles represent brain regions with significantly decreased nodal parameter after treatment.

For BC, after treatment, a significant increase was found in dFC_R, lat cerebellum_R, inf cerebellum_L, med cerebellum_R1; a significant decrease was found in occipital_L1, parietal_L1, parietal_L2, parietal_L3 and temporal_L. For DC, after treatment, a significant increase was found in precuneus_L, inf temporal_L, lat cerebellum_R, inf cerebellum_L, and med cerebellum_R1; a significant decrease was found in post cingulate_L, IPS_L, parietal_L2 and occipital_R1. For NCp, after treatment, we found a significant increase in aPFC_R; a significant decrease was found in mPFC, occipital_L2, mid insula_L, and med cerebellum_R2. For Ne, after treatment, a significant increase was found in inf temporal_L, lat cerebellum_R, inf cerebellum_L and med cerebellum_R1; a significant decrease was found in precuneus_L, post cingulate_L, IPS_L, vFC_R, parietal_L2, occipital_R1, occipital_R2. For NLe, after treatment, a significant decrease was found in precuneus_R, occipita_L2, med insula_L, med cerebellum_R2. For NLp, after treatment, a significant increase was found in precuneus_L, post cingulate_L, dlPFC_R, ACC_L, fusiform_R; a significant decrease was found in inf cerebellum_L, inf cerebellum_R (see Table 2 and Figure 2).

**TABLE 2:** Significant differences of node network parameters.

Network	Brain regions	*p*-values						MNI-coordinates		
		BC	DC	NCp	Ne	NLe	NLp	X (mm)	Y (mm)	Z (mm)
DMN	mPFC	0.412	0.657	↓0.042*	0.901	0.069	0.365	0	51	32
	Precuneus_L	0.123	↑0.002**	0.826	↓0.003**	0.58	↑0.002*	–3	–38	45
	Inf temporal_L	0.618	↑0.003**	0.145	↑0.002**	0.051	0.155	–61	–41	–2
	Post Cingulate_L	0.167	↓0.014*	0.887	↓0.022*	0.539	↑0.029*	–5	–52	17
	Precuneus_R	0.388	0.853	0.068	0.647	↓0.04*	0.398	11	–68	42
	IPS_L	0.076	↓0.04*	0.704	↓0.04*	0.739	0.419	–36	–69	40
	Occipital_L1	↓0.003**	0.445	0.722	0.364	0.616	0.358	–9	–72	41
	Occipital_L2	0.694	0.718	↓0.03*	0.571	↓0.03*	0.584	–42	–76	26
FPN	dlPFC_R	0.069	0.087	0.604	0.065	0.91	↑0.035*	40	36	29
	ACC_L	0.259	0.074	0.421	0.08	0.293	↑0.01*	–1	28	40
	dFC_R	↑0.031*	0.39	0.685	0.417	0.679	0.354	40	17	40
CON	aPFC_R	0.248	0.333	↑0.047*	0.345	0.05	0.433	27	49	26
	Med insula.L	0.705	0.219	↓0.04*	0.18	↓0.015*	0.132	–30	–14	1
	Fusiform_R	0.236	0.072	0.491	0.101	0.615	↑0.038*	54	–31	–18
	Parietal_L1	↓0.012*	0.348	0.306	0.276	0.544	0.587	–55	–44	30
SMN	vFC_R	0.063	0.057	0.329	↓0.048*	0.153	0.914	43	1	12
	Parietal_L2	0.074	↓0.017*	0.617	↓0.026*	0.291	0.206	–38	–15	59
	Parietal_L3	↓0.016*	0.054	0.885	0.067	0.732	0.066	–47	–18	50
	Parietal_L4	↓0.024*	0.507	0.101	0.497	0.156	0.343	–55	–22	38
	Temporal_L	↓0.008**	0.226	0.589	0.178	0.977	0.241	–54	–22	9
OCC	Occipital_R1	0.3	↓0.014*	0.828	↓0.028*	0.831	0.286	36	–60	–8
	Occipital_R2	0.906	0.054	0.645	↓0.047*	0.567	0.054	20	–78	–2
CBN	Lat cerebellum_R	↑0.025*	↑0.041*	0.894	↑0.039*	0.669	0.057	21	–64	–22
	Inf cerebellum_L	↑0.045*	↑0.019*	0.369	↑0.01*	0.526	↓0.022*	–34	–67	–29
	inf cerebellum_R	0.057	0.076	0.976	0.115	0.94	↓0.041*	33	–73	–30
	Med cerebellum_R1	↑0.011*	↑0.017*	0.161	↑0.008**	0.275	0.251	5	–75	–11
	Med cerebellum_R2	0.155	0.167	↓0.019*	0.125	↓0.043*	0.093	14	–75	–21

*BC, Betweenness Centrality, DC, Degree Centrality; NCp, Nodal Cluster Coefficients; Ne Nodal Efficiency; NLe, Nodal Local Efficiency; NLp, Nodal Shortest Path Lengths.*

*Represents a significance level of p < 0.05, ** represents a significance level of p < 0.01.

*↑Means that the topological property increases after treatment.*

*↓Means that the topological property decreases after treatment.*

*Values with p < 0.05 are bolded for better visualization.*

### Treatment Enhances the Betweenness Centrality of Fronto-Parietal Network

**FIGURE 3:**
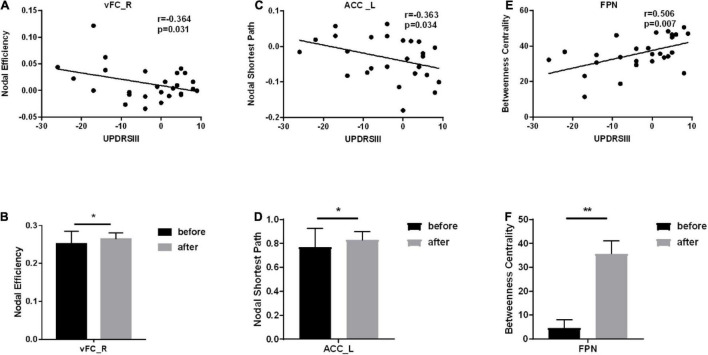
Brain regions that are significantly correlated with the scale and have different node parameters before vs. after treatment. *Represents a significance level of *p* < 0.05, ** represents a significance level of *p* < 0.01. **(A)** The correlation between the node efficiency of vFC_R and UPDRSIII. **(B)** The column chart of the node efficiency of VFC_ R before vs. after treatment. **(C)** The correlation between the node shortest path of ACC_L and UPDRSIII. **(D)** The column chart of the node shortest path of ACC_L before vs. after treatment. **(E)** The correlation between the betweenness centrality of FPN and UPDRSIII. **(F)** The column chart of the betweenness centrality ofFPN before vs. after treatment.

The nodal coefficients with significant differences were selected to perform Pearson correlation with UPDRSIII, thereby selecting brain regions with significant correlations. Among them, for Ne, there is a significant correlation between vFC_R of SMN and UPDRSIII. Significant correlation between ACC_L of FPN and UPDRSIII for NLp (see Figure 3).

To clarify the changes in each functional network, these brains were divided into six functional networks, including the CBN, CON, DMN, FPN, OCC, SMN. The results showed that for BC, the FPN network not only showed a significant improvement in treatment (*p* < 0.001), but also had a significant correlation with UPDRSIII. We draw a conclusion from this that treatment enhances the BC of FPN.

## Discussion

The main purpose of this study was to reveal changes in brain networks in PD patients after drug treatment from the perspective of functional connectivity and graph theory, and to explore the relationship between these features and motor symptoms. In our study, based on functional connectivity, there were significant differences in the 6 networks of the patients after treatment, of which 5 networks including CBN, DMN, FPN, OCC, SMN had obvious functional enhancement, while the functional connectivity of CON was weakened. We suspect it is the compensatory mechanism. In addition, important relevant functional connections are mainly concentrated in the CBN. For the nodal network coefficients, compared to before treatment there were also significant differences after treatment, which were mainly concentrated in the brain regions of the DMN and the CBN. It is worth mentioning that we have also done research on global network coefficients, including small worlds, network efficiency, and rich clubs, and there are no significant differences. For BC, the FPN network not only showed significant improvement after treatment (*p* < 0.001), but also had a significant correlation with the Motor Symptom Scale. Numerous studies have shown that functional connections between the FPN and other brain regions are reduced in patients with PD under rs-fMRI (Engels et al., 2018; Boon et al., 2020). Our study shows that treatment can effectively enhance functional connections between the FPN and other brain regions.

While traditional PD research has focused on basal ganglia dysfunction, this study supports a role for the cerebellum in PD. Previous studies have confirmed the presence of dopaminergic meridians and dopamine D1–3 receptors in the cerebellum (Wu and Hallett, 2013). The cerebellum receives dopaminergic projections from the ventral tegmental area/substantia nigra pars compacta. Differences in cerebellar activity may be considered a pathological mechanism related to basal ganglia dysfunction or a compensatory mechanism (Ballanger et al., 2008). The nature of cerebellar involvement is complex and may be influenced by dopamine, patient subtype, and specific symptoms or assessed function (Giompres and Delis, 2005). In this study, after treatment, functional connections that were significantly different and significantly correlated with UPDRSIII clustered in the cerebellum. Figure 1 visually shows that relevant functional connectivity is clustered in the cerebellum. Based on the findings, we can conclude that after treatment, there were significant changes in the connectivity of the brain functional network, and these changes were mainly related to the CBN. The typical tremor type in PD is resting tremor. Studies have shown that the cerebellum and/or its circuits play a crucial role in Parkinson’s tremors (Wu and Hallett, 2013).

Our understanding of the role of the cerebellum in PD is limited, and further research is needed to elucidate cerebellar pathology associated with PD and how cerebellar pathology and compensatory effects evolve as the disease progresses. A better understanding of the functional and morphological changes of the cerebellum associated with PD will have important implications for the pathophysiology of PD and may contribute to the development of new therapeutic strategies and targets.

Graph theory analysis methods have been widely used to analyze the structural and functional networks of brain magnetic resonance images (Bullmore and Sporns, 2009; Zhang and Luo, 2017; Ray et al., 2018). This method reflects some properties of the whole-brain network by quantifying the topological properties of each sub-network of the whole-brain (Fragkou et al., 2021). In graph theory, BC is one of the measures of the centrality of network graphs based on shortest paths. The shortest path length is one of the indicators to measure the information transmission ability in the brain, which can be used to evaluate the functional integration ability of the brain network. The shorter the length, the higher the functional integration ability. For a fully connected network graph, any two nodes have at least one shortest path. In an unweighted network graph, the shortest path is the sum of the number of paths that contain an edge, and in a weighted network graph, the shortest path is the sum of the weights of the paths that contain an edge (Chehreghani, 2014). The BC of each node is the number of times these shortest paths pass through that node.

Previous studies have demonstrated reduced functional connectivity of the FPN to other networks in PD. However, previous studies on the FPN mostly focused on executive function and cognitive function (Boon et al., 2020; Fathy et al., 2020). It has been suggested that the functional connectivity between FPN and SMN may reflect athletic performance. Prodoehl and Zhu’s study also showed that compared with tremor, non-tremor-dominant PD patients had decreased activity in the globus pallidus and the ipsilateral dorsolateral prefrontal cortex (a key player in the basal ganglia and FPN), respectively (Prodoehl et al., 2013; Zhu et al., 2021). Matsui study finds involvement of the parietal lobe associated with sensorimotor coordination impairment (Matsui et al., 2005). The cortex of the frontal and parietal lobes plays an important role in the development of movement as direct and indirect feedback channels for information processing. In the research related to rs-fMRI, patients with PD have reduced functional connectivity in the sensorimotor area, frontal and parietal networks (Lee et al., 2012; Baek et al., 2014), and Parkinson’s drug treatment can improve and enhance the functional connectivity of the FPN. In our study, the FC and DC of FPN were significantly enhanced after treatment (see Figures 1, 3). Anyway, FPN is very important for the treatment of PD (Engels et al., 2018). This study highlights the changes in the cerebellum and FPN during the treatment of PD, which is of great significance for people to further study the pathogenesis of the disease (Boon et al., 2020).

However, the study still has certain limitations. First, due to the incomplete website data, our sample size is small, which makes us ignore the influence of some special circumstances in the research process. Second, we still use traditional scales to assess symptoms, which are subject to the subjective judgment of experienced and trained physicians. It is possible to reduce the impact of this problem by using more objective symptom assessment methods. In my opinion, the following aspects can be considered in future research work: firstly, increase the sample size to enhance statistical reliability; secondly, use multiple batches of data to verify the results to avoid the chance of a single data; finally, combine the brain Electrogram data to explore brain changes in PD from a multimodal perspective (Hu and Zhang, 2020).

## Conclusion

For the analysis of functional connectivity, we conclude that there are significant changes in the connectivity of the brain functional network after treatment, and these changes are mainly related to the CBN. Combined with graph theory, our study provides valid evidence about the tight connection between motor symptoms and FPN networks in PD. These findings provide important implications for understanding the neural substrates underlying PD and suggest that FPN may serve as a new physiological biomarker.

## Data Availability Statement

The data analyzed in this study was obtained from the Parkinson’s Progression Marker Initiative (PPMI), the following licenses/restrictions apply: Investigators seeking access to PPMI data must sign the Data Use Agreement, submit an Online Application and comply with the study Publications Policy. Requests to access these datasets should be directed to PPMI (https://www.ppmi-info.org/access-data-specimens/download-data).

## Ethics Statement

The studies involving human participants were reviewed and approved by the Human Trials Ethics Committee. The patients/participants provided their written informed consent to participate in this study.

## Author Contributions

QL, TL, SF, and JZ conceived and designed the experiments. QL drew an illustration of the manuscript. QL, ZS, and KW performed the experiments. QL and JZ analyzed the data and wrote the article. All authors contributed to the article and approved the submitted version.

## Conflict of Interest

The authors declare that the research was conducted in the absence of any commercial or financial relationships that could be construed as a potential conflict of interest.

## Publisher’s Note

All claims expressed in this article are solely those of the authors and do not necessarily represent those of their affiliated organizations, or those of the publisher, the editors and the reviewers. Any product that may be evaluated in this article, or claim that may be made by its manufacturer, is not guaranteed or endorsed by the publisher.

## Footnotes

1
http://ppmi-info.org/study-design

2
http://www.nitrc.org/projects/GRETNA/

3
http://www.spss.com/
